# Human cornea conservation in coconut water solution

**DOI:** 10.5935/0004-2749.20210027

**Published:** 2025-02-02

**Authors:** David Antonio Camelo Cid, Marília Cavalcante Araújo, Márcia Vitorino Sampaio Passos, Ivelise Regina Canito Brasil, José Ferreira Nunes, Cristiane Clemente de Melo Salgueiro, Sávio Carvalho Nogueira, Dácio Carvalho Costa

**Affiliations:** 1 Hospital Geral de Fortaleza, Fortaleza, CE, Brazil; 2 Faculdade de Medicina, Universidade Estadual do Ceará, Fortaleza, CE, Brazil; 3 ACP Biotecnologia, Renorbio, Universidade Estadual do Ceará, Fortaleza, CE, Brazil; 4 Universidade Estadual do Ceará, Fortaleza, CE, Brazil

**Keywords:** Cornea, Coconut water, Organ preservation/methods, Organ preservation solution, Biotechnology, Córnea, Água de coco, Preservação de órgãos/métodos, Solução para preservação de órgãos, Biotecnologia

## Abstract

**Purpose:**

The aim of this study was to evaluate the physical and chemical characteristics of coconut water and to analyze the use of coconut water solution for the conservation of human corneas.

**Methods:**

This was an experimental and controlled study performed at the Eye Bank of the General Hospital of Fortaleza. The coconut water-based solution was prepared at the Goat Seed Technology Laboratory of the Department of Veterinary Medicine of the State University of Ceará. Discarded corneas from the Eye Bank were divided into two groups for sequential experiments: G1, coconut water-based solution (experimental group), and G2, conservative treatment with OPTISOL GS^®^ (control group). The osmolality of corneas in G1 was analyzed sequentially at 275, 300, 325, 345, 365, and 400 mOsm/L. The viability of the corneas was determined by specular microscopy and biomicroscopy on the first, third, and seventh days.

**Results:**

Corneas preserved in a solution of 365 and 345 mOsm/L had a transparency of 8 mm until the third day and had diffuse edema in the periphery, central folds, and partial epithelium loss until the seventh day. The 365-mOsm/L solution was associated with the worst results during follow-up. Corneas placed in Optisol-GS retained their original aspects.

**Conclusions:**

Coconut water-based preservative partially maintained corneal transparency and epithelial integrity, especially during the first three days of follow-up. The coconut water-based solutions used were not effective for use as preservatives in a human eye bank.

## Abstract (pt)

**Objetivos:**

As características físico-químicas e o baixo custo da água de coco foram fundamentais para o este estudo. Analisar o uso de solução a base de água de coco como meio de conservação de córneas humanas em banco de olhos.

**Métodos:**

Estudo experimental e controlado realizado no Banco de Olhos do Hospital Geral de Fortaleza. Utilizou-se solução à base de água de coco preparada no laboratório de Tecnologia de Sêmen de Caprinos do Departamento de Medicina Veterinária da Universidade Estadual do Ceará. Foram usadas córneas de descartes divididas em dois grupos: G1 (Conservante com água de coco) - grupo experimental e G2 (grupo Conservante com OPTISOL GS^®^) grupo controle, em experimentos sequenciais. A osmolaridade do G1 foi analisada sequencialmente com 275, 300, 325, 345, 365 e 400 mOsm/L. A viabilidade das córneas foram realizadas por microscopia especular e biomicroscopia nos 1^o^, 3^o^ e 7^o^ dias.

**Resultados:**

As córneas em solução de 365 e 345 mOsm/L apresentavam transparência nos 8mm centrais até o 3^o^ dia, com edema em toda periferia, dobras centrais e edema 2+, com perda parcial do epitélio até 7^o^ dia, sendo o de maior osmolaridade com melhor transparência durante o seguimento. Grupo com 275, 300 e 400 mOsm/L, córnea opaca, edema difuso, perda total do epitélio no 3^o^ dia. As córneas em Optisol mantiveram seus aspectos.

**Conclusões:**

O conservante à base de água de coco manteve em parte a transparência corneana e a integridade epitelial, especialmente nos primeiros 3 dias de seguimento. A solução conservante com água de coco nas formulações utilizadas não se mostrou eficaz para o uso em banco de olhos humanos.

## INTRODUCTION

The cornea has a complex structure and has important optical and protective roles.^(1)^. Its immunologically privileged nature results in a low graft rejection index after transplantation^(2)^, making corneal transplant the most successful procedure among all human tissues transplants^(3)^, with an overall 5-year graft survival greater than 65%^(4)^.

The human cornea endothelium is a monolayer of hexagonal cells, which are responsible for the maintenance of corneal transparency^(5)^. The function of the endothelium can be estimated *in vivo* by evaluating the corneal thickness (pachymetry) and morphological surface integrity using specular microscopy^(5)^.

In 1953, Stocker demonstrated the importance of cor neal endothelium in maintaining the transparency of the cornea^(6)^. After this observation, corneal preservation methods were focused on maintenance of endothelial function and morphological integrity^(7)^.

Until the mid-1970s, transplanted corneas were removed from the living donor’s enucleated eyes. Subsequently, with the advent of M-K^®^ (McCarey-Kaufman) media, it became possible to preserve donor tissue for three or four days at 4°C. Preservation media such as Optisol GS^®^ are now available, which keep the tissue viable for approximately two weeks at 4°C^(8)^.

In the 1940s, Professor Van Overbeek et al. demonstrated that coconut water, when used as a culture medium, had the potential to improve the growth of cells, tissues, and organs *in vitro*^(9)^. These results were also corroborated in another study, which coconut water was used as a goat sperm preservative^(10-12)^.

In the 1990s, a low-cost coconut water solution was demonstrated *in vitro* when used as a preservative culture medium for rabbit corneas, capable of maintaining the deturgence and conservation of the corneal epithelium and endothelium^(13)^. The excellent results of this experimental study were decisive in choosing the media solution for the present work.

To evaluate of these aspects, and considering the physicochemical characteristics and the low cost of coconut water, the aim of this study is to analyze the potential use a coconut water-based solution as a medium for human cornea conservation.

## METHODS

This present experimental study was performed at the Banco de Olhos do Hospital Geral de Fortaleza from April 2017 to February 2018. Coconut water was prepared at the Goat Semen Technology Laboratory of Ceará’s State University Department of Veterinary Medicine.

We collected corneas not eligible for transplant (discarded corneas) but without any changes related to endothelial dysfunction from the hospital’s Eye Bank. Samples were matched from the same donor and randomly assigned to control and experimental groups, one with OPTISOL-GS^®^ media and the other with coconut water media, respectively.

### Evaluation of discarded corneas

Corneal evaluation was performed by the same researcher using biomicroscopy on days 0, 1, 3, and 7, taking into account the degree of exposure and epithelial defect, subepithelial opacity, edema, stria, and stromal infiltrate, Descemet folds, loss of endothelial cells, endothelial guttata, pterygium, senile arch, scars, and specular reflex. Each parameter was classified by scores ranging from 0 to 4, with 0 being excellent; 1, good; 2, regular; 3, bad; and 4, unacceptable. Findings from specular microscopy (CellChek D, Konan Medical, Irvine, CA, USA) were also evaluated on days 0, 3, and 7.

### Coconut water-based solution

For the coconut water-based solution, we used coconut water of the dwarf variety on the fifth month of maturation, with an average pH of 7.1, plus gentamicin 200 µg/mL, 2.5% chondroitin sulfate, 1% dextran, balanced saline and buffer solution, with variable osmolarity.

We used ACP-412 powdered coconut water used, whose composition is shown in Chart 1. ACP-412 medium was resuspended in Milli-Q water and physically sterilized using a 0.22-µm Millipore filter (Microlab Scientific, China). The solution resulting from this process was stored in sterile plastic vials and cooled to 4°C. During the preparation and handling of the corneas, temperatures did not exceed 18°C. Solutions were prepared at different osmolarities (275, 300, 325, 345, 365, 400 mOsm/L).

**Chart 1:** Biochemichal composition of powdered coconut water (ACP 412).

		Vitamin	
		Vitamin B1 (mg),	0.17
		. Thiamine	
Calories (kcal)	378	Vitamin B3 (mg), Niacin	0.12
Calories (KJ)	1585	(Nicotinic acid and	
		vitamin PP)	
Carbohydrates (kcal)	372		
		Vitamin B5 (mg),	6.51
		
Carbohydrates (g)	93.00	pantothenic acid	
Fructose (g)	50.02	Vitamin B12 (mcg),	0.22
Galactose (g)	0.00	Cobalamin	
Glucose (g)	34.97	Folic acid (mcg)	312.00
		Vitamin C (mg), Ascorbic	26.80
Protein (g)	0.90	acid	
Total fats (g)	0.30	Vitamin D (mcg)	1.50
Saturated fats (g)	0.00	Calciferol	
Monounsatured fats (g)	0.00	Biotin	8.03
		Amino acids	
Poly satured fats (g)	0.00	Aspartic acid (mg)	
Trans fat (g)	0.00		0.70
		Glutamic acid (mg)	172.00
Cholesterol (g)	0.00		
		Alanine (mg)	38.60
Total fibers (g)	4.30	Arginine (mg)	126.00
		
Fibers (g)	-	Cystine (mg)	14.80
Insuluble dietary fibers (g)	4.10	Phenylalanine (mg)	38.00
Soluble dietary fibers (g)	0.20	Glycine (mg)	36.40
Humidity (g)	3.00	Glutamine (mg)	172.00
Ashes (g)	1.30	Histidine (mg)	17.80
Total solids (g)	97.01	Isoleucine (mg)	29.30
Minerals		Leucine (mg)	54.20
Sodium (mg)	105.000	Lysine (mg)	33.10
		Methionine (mg)	14.00
Calcium (mg)	39.000		
		Proline (mg)	32.00
Iron (mg)	0.30		
		Serine (mg)	39.00
Phosphorus (mg)	45.200	Tyrosine (mg)	24.00
Magnesium (mg)	25.000	Threonine (mg)	28.20
Manganese (mg)	1.100	Triptophan (mg)	8.40
Potassium (mg)	250.00	Valine (mg)	48.00

Source: ACP Biotecnologia (2017).

### Interpretation of results and statistical analysis

Data were analyzed and discussed with the selected bibliography, using tables and graphs when necessary. The Kolmogorov-Sminorv test was used to evaluate the distribution of groups G1 and G2, and Student *t* test was used for two independent populations to determine the differences between groups G1 and G2. For all statistical tests performed in the present study, we adopted p<0.05 as the significance level.

### Ethical considerations

The project was approved by the Research Ethics Committee of the Fortaleza General Hospital (HGF), CAAE: 65797717.3.0000.5040. Researchers followed the Declaration of Helsinki and Resolution 196/96 of the National Health Council during both the preparation and execution of the study.

The corneas used in the study were discarded; therefore, there was no effect on the transplant waiting list and no risk of contamination for the researchers, even when serologies were positive.

## RESULTS

During the period, 28 corneas were selected. We analyzed the corneeas in pairs, with one cornea belonging to G1 (coconut water) and the other to G2 (Optisol GS^®^), which had a pH of 7.30 and an osmolarity of 365 mOsm/L. The G1 medium consisted of several solutions with different osmolarities (mOsm/L) and different temperatures, whereas the G2 medium group had the same osmolarity and temperature. There was no statistically significant between-group difference in vitality parameters (Table 1) as measured by biomicroscopy; mean donor age; or specular microscopy data before the experiments.

**Table 1:** Comparison between groups G1 and G2 before the experiments

	G1	G2	t	p-value^*^
Average score biomicroscopy (points)	1.5	1.33	0.69	**0.49**
Average age of donor (years)	40.67	40.67	0.02	**0.98**
Specular microscopy CD cel/mm^2^	2039	2053	0.33	**0.74**
CV (%)	40	39	0.09	**0.91**
HEX (%)	42	43	0.31	**0.75**

* Student *t* test.

### Experiment 1

**Figure 1:**
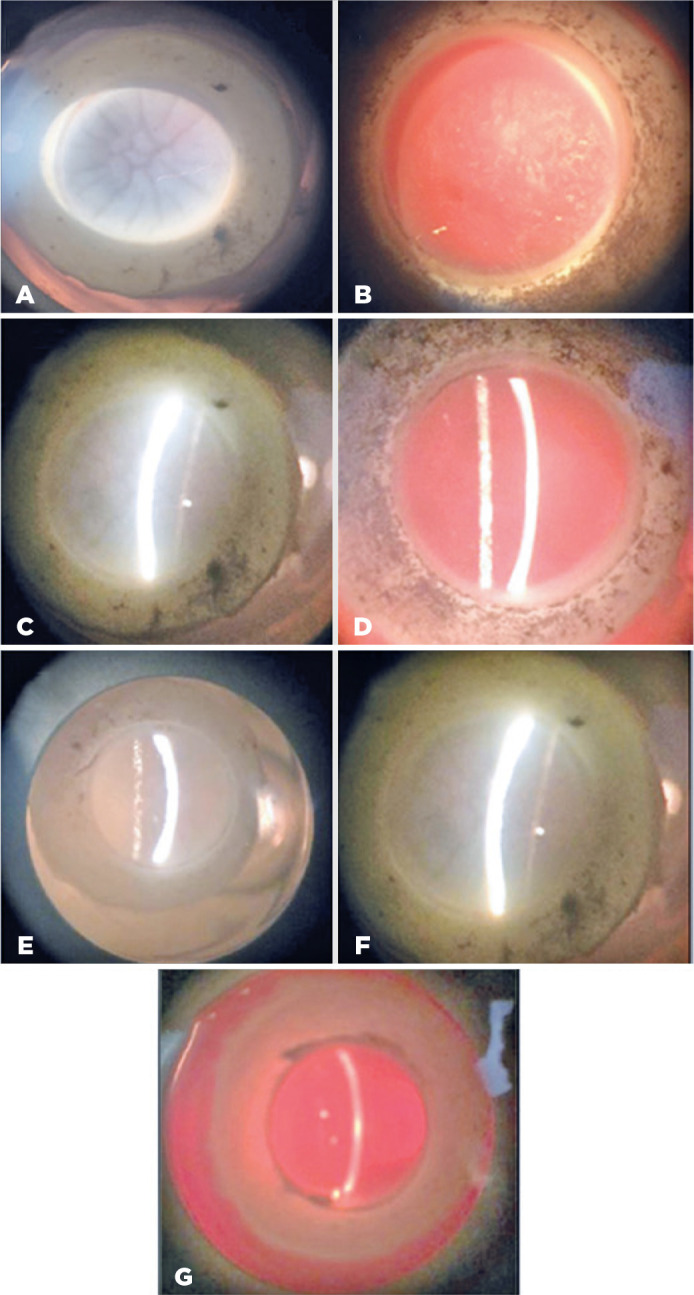
(A) G1 (coconut water) 300 mOsm/L. (B) G2 (Optisol GS) 360 mOsm/L. (C) G1 (coconut water) 345 mOsm/L. (D) G2 (Optisol GS) 365 mOsm/L. (E) G1 (coconut water) 400 mOsm/L. (F) G1 (coconut water) 250 mOsm/L. (G) G2 (Optisol GS) 360 mOsm/L. All specimens after three days in solution.

In the first experiment, we tested two paired corneas. The G1 and G2 media had, respectively, an osmolarity of 300 and 365 mOsm/L and a pH of 7.25 and 7.30. All media were maintained at temperatures between 2 and 4°C. After three days in the preservatives, the G1 corneas had 4+/4+ stromal edema, marked Descemet membrane folds, and loss of the entire epithelium (Figure 1A). The G2 results were unchanged (Figure 1B).

### Experiment 2

In the second experiment, G1 had an osmolarity of 345 mOsm/L and a pH of 7.32. The results in G2 remained unchanged. On the experiment’s third day, the G1 corneas had 3+/4+ stromal edema with moderate Descemet membrane folds and partial epithelium loss (Figure 1C). An endothelial count was impossible to perform.

### Experiment 3

We evaluated how G1 would react with two osmolarities between the extremes for human corneal conservation, 250 and 400 mOsm/L, one tending toward hypo-osmolarity and the other to hyperosmolarity (pH 7.12 and 7.28, respectively).

G1 had 4+/4+ edema, especially in the group with an osmolarity of 250 mOsm/L (Figure 1F). The lower os molarity group had loss of the entire corneal epithelium and pronounced folds. Specular microscopy was impossible to perform. Corneas in G2 did not demonstrate changes in either their structure or emergence of stromal edema (Figure 1G).

### Experiment 4

**Figure 2:**
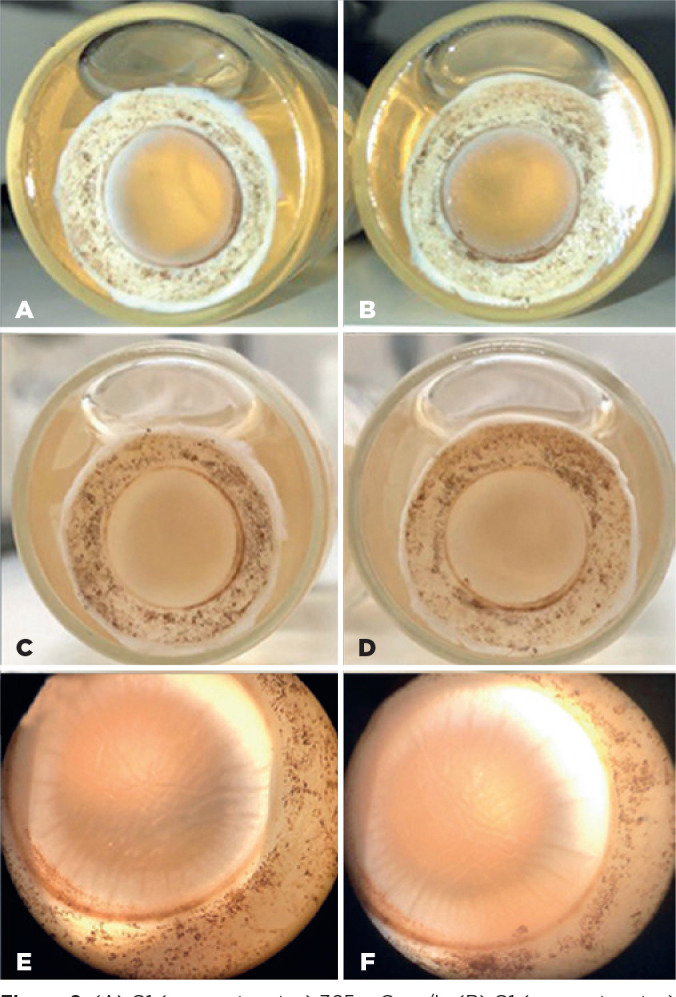
(A) G1 (coconut water) 365 mOsm/L. (B) G1 (coconut water) 345 mOsm/L. (C) G1 (coconut water) 365 mOsm/L. (D) G1 (coconut water) 345 mOsm/L. (E) G1 (coconut water) 365 mOsm/L. (F) G1 (coconut water) 345 mOsm/L. Solutions on the third day.

**Figure 3:**
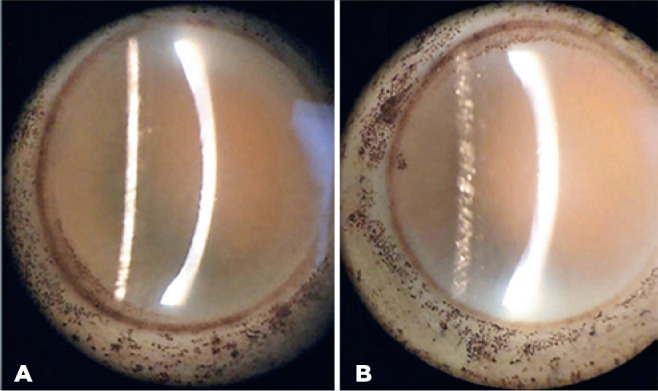
(A) G1 (coconut water) 365 mOsm/L. (B) G1 (coconut water) 345 mOsm/L. Solutions on the seventh day.

For this experiment, we decided to perform temperature-controlled baths at 18°C, and two corneas were tested in 365 and 345 mOsm/L media. The results of the experiements were as follows:

- Day 3: Weakened corneal transparency, marked loss of epithelium, and most significant diffuse stromal edema in the 345 mOsm/L sample (Figure 2).
- Day 7: Cornea with subtotal loss of transparency, loose epithelium, and marked diffuse stromal edema in both media (Figure 3).

### Experiment 5

**Figure 4:**
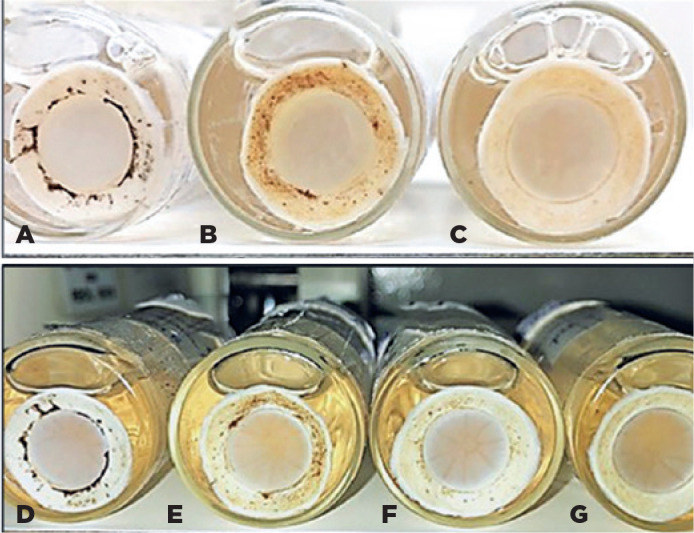
(A) G1 (coconut water) 325 mOsm/L. (B) G1 (coconut water) 345 mOsm/L. (C) G1 (coconut water) 365 mOsm/L. Solutions on the seventh day. (D) G1 (coconut water) 275 mOsm/L. (E) G1 (coconut water) 325 mOsm/L. (F) G1 (coconut water) 345 mOsm/L. (G) G1 (coconut water) 365 mOsm/L.

There were three corneas in G1 at 325, 345, and 365 mOsm/L, presenting the following results:

- Day 3: Opaque cornea, diffuse edema in the 325 mOsm/L group; the 365 mOsm/L group maintained better transparency and lower stromal edema between the three tests, with partial epithelial loss in the central region with an 8-mm diameter.

- Day 7: Accentuated corneal edema in all groups; partial loss of epithelium in the 345 and 365 mOsm/L groups (Figure 4B, C).

### Experiment 6

There were four corneas in the 275, 325, 345, and 365 mOsm/L groups, presenting the following results:

- Day 3: Opaque cornea, diffuse edema in the 275, 325, and 345 mOsm/L groups; The 275 mOsm/L group had worse edema and coarse folds, and the 365 mOsm/L group had better transparency and lower stromal edema between the three tests, with partial epithelium loss (Figure 4G).
- Day 7: Accentuated corneal edema in all groups.

## DISCUSSION

Coconut water has been described in several studies as an alternative tissue conservation product, and it was chosen as the nutritive medium for the preservative solution in question because of its physicochemical charac teristics, low, cost and abundance in our region.

Since the 1980s, its use as a natural preservative has been researched during experiments with semen and embryos of sheep and goats^(10,11)^. Later on, in a study with rabbit corneas using a coconut water-based solution, no significant structural change was observed in relation to the corneas preserved in Optisol^®(13)^.

We observed progressive loss of the corneal epithelium on all conservation days analyzed, especially in media with extreme osmolarity (275 and 400 mOsm/L). A study that used a similar solution in feline corneas presented medium osmolarity with coconut water of 305 mOsm/L, which was lower than Optisol-GS^®^ (360 mOsm/L). This was decisive in the negative outcome of the study. Tachibana et al.^(14)^ demonstrated that Optisol-GS^®^ to maintain transparency and prevent stromal edema occurs because of its high osmolarity. The worst results found in our first experiments were most likely related to incorrect osmolarity. Significant improvement in edema and thus corneal transparency was detected after adjusting the solution to 365 mOsm/L, which is equivalent to that found in the Optisol GS^®^ standard solution.

With regard to the pH, the medium tested was adjusted at a pH of about 7.12, with few variations between the prepared solutions (pH 7.12-7.30). Previous studies have reported that the corneal endothelium tolerates a pH variation between 6.5 and 8.5 and that media formulated with pH within this range are acceptable^(14)^. Thus, we believe that the pH had no influence on the final results of the study.

The corneal endothelium is very sensitive at low temperatures, and when it rests in liquid media at 4°C, free radical formation may occur. This may further induce cellular apoptosis^(15)^, leading to structural membrane changes with important consequences on the inhibition of the metabolism^(16)^. Thus, after the first results, we decided to dilute the solution as well preserve the cornea under strict temperature control conditions so that no thermal shock or any damage to the endothelium could occur. The corneal conservation occurred at a temperature between 2°C and 8°C in the media we used and did not exceed 18°C during the handling of the corneas.

In their experimental study in rabbits, Nogueira et al.^(13)^ used coconut water as a culture medium in corneal preservatives in an experimental study in rabbits. These authors suggested that coconut water has properties that maintain corneal epithelium as well as endothelial deturgence and conservation in vitro. The excellent results of that study resulted in our decision to use this solution for human corneas. However, our results did not corroborate the findings of that previous study, with the 365 mOsm/L osmolarity solution having the best ability to maintain corneal transparency and partially intact epithelium within the first three days.

Rabbit eyes are believed to have great anatomical and histological similarity to human and domestic animal eyes and have therefore been used extensively in experimental medical studies. However, several corneal structural differences have been observed among domestic animals. The cornea of nocturnal animals can comprise about 35% of the bulb surface, whereas that of domestic animals can range from 17% to 30%. Furthermore, the thickness of the cornea varies between species, ranging from 0.5 to 1 mm. In addition, the Bowman layer is not seen in most animals and is generally described only in humans and nonhuman primates. There is controversy regarding the ability of the epithelium to regenerate, and in fact, it may vary among different species and ages. In general, however, some mitotic activity may occur mainly in young animals^(17)^. It is therefore possible that such structural differences may have interfered with the results of the study, with the human cornea being perhaps less resistant to variations in osmolarity, temperature, and pH or having a reduced regenerative capacity as compared with rabbit cornea. Further studies using the ACP-412 solution in rabbit corneas, similar to that performed in the 1990s^(13)^, are required to clarify these hypotheses.

In the study by Nogueira et al.^(13)^, the final preservative solution was obtained by mixing 33% coconut water prepared according to Nunes, 33% balanced saline, 33% of 1% Dextran in ringer lactate, and 2.5% chondroitin sulfate. The pH was changed to 7.4, and the osmolarity was maintained at 300 mOsm/L. The final product was filtered through a Millipore filter and distributed over sterile vials. Therefore, its powdered form was not used, and the ideal osmolarity was determined as 300 mOsm/L. It is possible that this fact interfered with the outcome of this study. We encountered difficulties related to product dilution and in particular during filtering through the Millipore filters, which consistently became clogged during the procedure.

Several studies have demonstrated the effectiveness of powdered coconut water, especially in animal reproduction experiments. Standardization of powder products have enabled the maintenance of their physical, chemical, and biological properties, reducing conservation-related biases^(11)^. In 2002, this standardization was successfully achieved by processing the liquid and transforming it into powder^(18)^.

In contrast, the results obtained in this study indicate that coconut water did not serve as a nutritive medium for human corneas because of the morphological changes observed in the corneal epithelium and endothelium via specular microscopy and slit lamp biomicroscopy.

Coconut water-based organ and tissue preservatives are very promising biotechnological products. For this reason, broader studies should be performed with human corneas to obtain an ideal solution with low cost and the ability to maintain the integrity of corneal tissue.

Our results show that the coconut water-based solution partly retained corneal transparency and epithelial integrity, especially in the first three days of follow-up, but specular microscopy was not possible. The best results were achieved with a solution with 365-mOsm/L osmolarity. However, the preservative solution with coconut water was not effective for human eye bank use. The results of this study did not corroborate the findings of previous research performed in rabbit corneas.
